# A new paradigm in modelling the evolution of a stand via the distribution of tree sizes

**DOI:** 10.1038/s41598-017-16100-2

**Published:** 2017-11-20

**Authors:** Petras Rupšys, Edmundas Petrauskas

**Affiliations:** 1 Institute of Forest Management and Wood Sciences, Aleksandras Stulginskis University, Studentu 11, Kaunas, LT-53361 Lithuania; 2 Centre of Mathematics, Physics and Information Technologies, Aleksandras Stulginskis University, Studentu 11, Kaunas, LT-53361 Lithuania

## Abstract

Our study focusses on investigating a modern modelling paradigm, a bivariate stochastic process, that allows us to link individual tree variables with growth and yield stand attributes. In this paper, our aim is to introduce the mathematics of mixed effect parameters in a bivariate stochastic differential equation and to describe how such a model can be used to aid our understanding of the bivariate height and diameter distribution in a stand using a large dataset provided by the Lithuanian National Forest Inventory (LNFI). We examine tree height and diameter evolution with a Vasicek-type bivariate stochastic differential equation and mixed effect parameters. It is focused on demonstrating how new developed bivariate conditional probability density functions allowed us to calculate the evolution, in the forward and backward directions, of the mean diameter, height, dominant height, assortments, stem volume of a stand and uncertainties in these attributes for a given stand age. We estimate the parameters by considering discrete samples of the diameter and height at a given age and by using an approximated maximum likelihood procedure. The model performance criteria for the height and diameter growth models include statistical indexes and an analysis of residuals.

## Introduction

Foresters seek to predict patterns in the distributions of individual trees within stands, across tree sizes and over space. Determining a distribution of tree diameters and heights has been broadly studied since the twentieth century^1^. Originally, the tree diameter and height distribution concept crystallised in assessing standing timber value based on merchantable piece sizes and forest structure which are the basis of aboveground forest carbon estimates^2^. The distribution of tree diameters at the breast height (in the sequel – diameter) is one of the most investigated subjects of statistical research in forestry. The forestry literature reports that tree diameter distributions vary across different stands^3^. In forest research, the univariate tree diameter distribution was introduced by Meyer^4^ and Schnur^5^ using a discrete probability space methodology. Natural extensions of a discrete diameter distribution are the continuous univariate gamma distribution^6^, log-normal distribution^7^, Weibull distribution^8^, logit-logistic^9^ among others. Classical mathematical modelling of tree size distribution is largely concerned with the use of a well-known probability density function for fitting to the empirical tree size frequencies and does not quantify the evolution of a tree size distribution via a forest stand age. Several authors have analysed the tree height distribution in a forest stand. The tree height distribution can be depicted using two mathematical models. First, a height distribution can be derived indirectly from a diameter distribution together with the relationship between tree height and diameter. The relationship between height and diameter distributions can be accounted, provided that the function relating to height and diameter can be inverted^10^. Second, theoretical height distributions were fitted directly to tree height observations and have included such shapes as the beta^11^, the Weibull^8^, the Johnson^12^, the power-normal^13^ and much more. The construction and applications of bivariate distributions of diameter and height are the most used classical fields of research in forest statistics, and it continues to be an active field of research in forest mensuration and inventory, where both the diameter and height are measured on every tree.

The philosophy of the mean stem volume estimation is based on the modelling of the distributions of the volume components of diameter and height instead of the volume itself. The bivariate distribution, as a possible relevant model for mean stem volume statistics, provides more physical insights on the validity domain and the quantification of the error made by using such a distribution. A bivariate distribution of diameter and height can be developed using several mathematical techniques. First, a theoretical diameter and height distribution can be fitted directly to tree diameter and height observations in forms such as the Johnson^2,14^, the power-normal^15^ and others. Second, a theoretical bivariate diameter and height distribution as an extension of univariate marginal distributions can be developed by Plackett’s method^16,17^, the copula method^18^ and references therein.

A new paradigm in stand structure, modelling beyond well-defined theoretical distributions, is to use stochastic differential equations (SDEs) techniques. On the other hand, an algebraic structure of SDEs can relate tree size distribution and the age of a forest stand. Nowadays, SDEs find applications in many disciplines including physics^19^, information^20^, biomedicine^21^ and forestry^22^. The framework of SDEs can also be adapted to modelling theoretical diameter and height distributions^23^. One motivation for using SDEs for the modelling of the evolution of the tree size distribution is that unknown drivers functioning in a stand can be modelled as random processes, which lead to a white noise process. The solution to the SDE of the diameter and height dynamics is a bivariate random process which leads to a theoretical bivariate probability density function for the diameter and height. In this philosophy, the evolution of the probability density function for diameter and height, which behaves according to a stochastic differential equation, can be defined by the Fokker-Planck equation. Some models describing the evolution of the fixed effect parameters univariate and bivariate probability density functions were discussed by Rupšys and Petrauskas^22,23^. Recently, there were also a number of results on mixed effect parameters using the Ornstein-Uhlenbeck family of univariate SDE models of tree height evolution via diameter. Such models generate the normal, or log-normal, probability density functions for diameter and height in a univariate sense^24–27^ or using the normal copula technique in a bivariate sense^28^.

The newly developed model based on bivariate mixed effects SDE extend the usual non-linear mixed effects regression models through the inclusion of a system noise as an additional source of variation in the first-stage model. This extended model describes the variation in the data through two sources of noise: the system noise, σ, which reflects the random fluctuations around the corresponding theoretical diameter and height models and represents the within-stand variation, and random effect, ϕ, which represents the between-stand variation in the theoretical diameter and height models. If the magnitude of the parameter of the system noise σ is zero, the entire system noise term will vanish, and the remaining part of the SDE will simply be the differential form, the solution of which is the regression term of the mixed effects model. Random effect, ϕ, is not parameter but it is a random variable. The distribution of random effect captures random variation of the parameter in the population of stands and is frequently assumed to be normal.

Nonlinear mixed effect regression models were introduced into forestry management to analyse data from several stands simultaneously. The individual tree size variables are assumed to be described by a common structural model with some of the model parameters varying between the stands (so-called random effects), while other parameters are invariant between stands (so-called fixed effect parameters). The mixed effects methodology can be of great benefit when the model uses the data of the National Forest Inventory as it is regional and sparse.

In this study, the ultimate objective is to build in some detail the bivariate Vasicek type mixed effect parameters SDEs for the diameter and height at a given stand age to deduce the evolution, via stand age, of the bivariate conditional probability density function. This can also be used for predicting stand characteristics (mean tree diameter, height, stem volume, stand volume per ha) and their precisions. The technique for estimating mixed effect parameters is based on an approximation of the maximum log-likelihood function. This work aims to present principal concepts of the newly developed bivariate mixed effect parameters conditional probability density function of the diameter and height in simple terms and to illustrate results using large datasets provided by the Lithuanian National Forest Inventory. All results are implemented in the symbolic algebra system MAPLE.

## Materials and Methods

### Data

The data used for the newly developed model estimation and validation were obtained from the Lithuanian National Forest Inventory (LNFI) (2007–2010). At plot establishment, the following data were recorded for every sample tree: the species, the age, the diameter over bark at 1.30 m height and measured to the nearest millimetre. The total tree height was measured for a subsample of trees (no less than 3) to the nearest quarter metre. The tree diameters were measured with field callipers in two perpendicular directions. In the main 500 m^2^ size circular plot (horizontal radius equals to 12.62 m) all larger than 14.0 cm in diameter trees at 1.3 m height were inventoried. A total of 2,108 plots of Scots pine trees were chosen from the LNFI 2007–2010 database. The dataset was randomly divided into estimation and validation datasets. A random sample of 1,408 plots (5,220 trees) was selected for model estimation, and the remaining dataset of 700 plots (2,692 trees) was utilised for model validation. Only measurements from live trees without top damage were included in the statistical analysis. Summary statistics for the diameter at breast height (d), the total height (h) and the age (A) for all of the trees used in model estimation and validation datasets are presented in Table 1. It should be noted that data on the number of plots with greater than 10 measured trees (diameter and height) were very limited.

**Table 1:** Datasets summary statistics.

Data	Number of trees	Min	Max	Mean	St. Dev.	Number of trees	Min	Max	Mean	St. Dev.
**Estimation**						**Validation**				
**d (cm)**	5220	15.10	66.10	27.24	8.63	2692	15.10	74.30	27.45	8.83
**h (m)**	5220	7.10	37.80	22.07	4.42	2692	7.2	34.80	22.26	4.43
**A (yr)**	5220	17	221	65.78	23.67	2692	17	162	67.70	23.31

### Bivariate stochastic differential equation framework

In this paper, we take a bivariate Ornstein-Uhlenbeck family^29^ SDE of the Vasicek type^22^ to study the tree diameter and height bivariate distribution problem. This results in an exact bivariate conditional probability density function which parameters can be estimated by maximum likelihood procedure based on discrete time observations. A bivariate mixed effect parameters SDE is set up for describing the tree diameter and height dynamics that extends to a theory of a bivariate conditional probability density function of the tree diameter and height. Proceeding as we have in the fixed effect parameters Gompertz-type bivariate case^23^, the Vasicek type bivariate SDE that describes the development of the diameter and height is defined by:

1 $$d{X}^{i}(t)=A({X}^{i}(t))dt+{B}^{\frac{1}{2}}\cdot dW(t),\,i=1,2,\ldots ,M,$$

here: M is the total number of stands used for model fitting, t is the time (stand age), *X*(*t*) = (*D*(*t*), *H*(*t*))^*T*^, *t*∈[*t*_0_; *T*], *t*_0_ ≥ 0, *X*(*t*_0_) = *x*_0_ = (*d*_0_, *h*_0_)^*T*^, *d*_0_ ≥ 0, *h*_0_ ≥ 0, the drift vector *A*(*x*) is defined as:

2 $$A(x)={({\beta }_{d}({\alpha }_{d}+{\varphi }_{d}^{i}-d),{\beta }_{h}({\alpha }_{h}+{\varphi }_{h}^{i}-h))}^{T},$$

the diffusion matrix B is defined as:

3 $$B=[\begin{array}{ll}{\sigma }_{11} & {\sigma }_{12}\\ {\sigma }_{12} & {\sigma }_{22}\end{array}],$$

${W}^{i}(t)={({W}_{d}^{i}(t),{W}_{h}^{i}(t))}^{T}$, *t*∈[*t*_0_; *T*] are independent bivariate Brownian motions, ${\varphi }_{d}^{i}$ and ${\varphi }_{h}^{i}$, *i* = 1, 2, …, M, are independent and normally distributed random variables with zero mean and constant variances (${\varphi }_{d}^{i} \sim N(0;{\sigma }_{d}^{2})$ and ${\varphi }_{h}^{i} \sim N(0;{\sigma }_{h}^{2})$), *α*_*d*_, *α*_*h*_, *β*_*d*_, *β*_*h*_, *σ*_11_, *σ*_12_, *σ*_22_, *σ*_*d*_, *σ*_*h*_ are fixed effect parameters to be estimated and *W* ^*i*^(*t*), *W* ^*j*^(*t*), ${\varphi }_{d}^{i}$, ${\varphi }_{d}^{j}$, ${\varphi }_{h}^{i}$ and ${\varphi }_{h}^{j}$ are mutually independent for all 1 ≤ *i*, *j* ≤ *M*. In the sequel, initial condition takes the form: if t_0_ = 5, then d_0_ = 0.0 and h_0_ = 1.3.

The Vasicek-type bivariate SDE can be converted into a well-studied bivariate Ornstein-Uhlenbeck^29^ process by the transformation $Y(t)={({e}^{{\beta }_{d}(t)}\cdot D(t),{e}^{{\beta }_{h}(t)}\cdot H(t))}^{T}$ and solved explicitly. By applying the Ito formula^30^ and taking into account that the random vector ${({\int }_{{t}_{0}}^{t}{e}^{{\beta }_{d}(t)}\cdot {W}_{d}^{i}(t),{\int }_{{t}_{0}}^{t}{e}^{{\beta }_{h}(t)}\cdot {W}_{h}^{i}(t))}^{T}$ has a bivariate normal distribution, we can deduce that the conditional random vector (*X* ^*i*^(*t*)|*X*(*t*_0_) = *x*_0_) = (*D*(*t*)|*D*(*t*_0_) = *d*_0_, *H*(*t*)|*H*(*t*_0_) = *h*_0_))^*T*^ has a bivariate normal distribution *N*_2_(*μ* ^*i*^(*t*); Σ(*t*)), *i* = 1, 2, … M, with the mean vector ${\mu }^{i}(t)={({\mu }_{d}^{i}(t),{\mu }_{h}^{i}(t))}^{T}$:

4 $${\mu }_{d}^{i}(t)={\alpha }_{d}+{\varphi }_{d}^{i}-({d}_{0}-({\alpha }_{d}+{\varphi }_{d}^{i}))\cdot {e}^{-{\beta }_{d}(t-{t}_{0})},$$

5 $${\mu }_{h}^{i}(t)={\alpha }_{h}+{\varphi }_{h}^{i}-({h}_{0}-({\alpha }_{h}+{\varphi }_{h}^{i}))\cdot {e}^{-{\beta }_{h}(t-{t}_{0})},$$

the variance-covariance matrix Σ(*t*):

6 $${\rm{\Sigma }}(t)=[\begin{array}{cc}{v}_{d}^{2}(t) & {v}_{d,h}^{2}(t)\\ {v}_{d,h}^{2}(t) & {v}_{h}^{2}(t)\end{array}],$$

7 $${\nu }_{d}^{2}(t)=\frac{{\sigma }_{11}(1-{e}^{-2{\beta }_{d}(t-{t}_{0})})}{2{\beta }_{d}},$$

8 $${\nu }_{h}^{2}(t)=\frac{{\sigma }_{22}(1-{e}^{-2{\beta }_{h}(t-{t}_{0})})}{2{\beta }_{h}},$$

9 $${\nu }_{d,h}^{2}(t)=\frac{{\sigma }_{12}(1-{e}^{-({\beta }_{d}+{\beta }_{h})(t-{t}_{0})})}{{\beta }_{d}+{\beta }_{h}},$$

and conditional probability density function:

10 $$f(d,h,t|{\alpha }_{d},{\alpha }_{h},{\beta }_{d},{\beta }_{h},{\sigma }_{11},{\sigma }_{12},{\sigma }_{22},{\sigma }_{d},{\sigma }_{h},{\varphi }_{d}^{i},{\varphi }_{h}^{i})=\frac{1}{2\pi dh{[{\rm{\Sigma }}(t)]}^{-1}}\exp (-\frac{1}{2}{\rm{\Omega }}(d,h,t)),$$

11 $${\rm{\Omega }}(d,h,t)=(d-{\mu }_{d}^{i}(t),h-{\mu }_{h}^{i}(t)){[{\rm{\Sigma }}(t)]}^{-1}(\begin{array}{c}d-{\mu }_{d}^{i}(t)\\ h-{\mu }_{h}^{i}(t)\end{array}).$$

## Results and Discussion

The conceptually simplest modelling methodology for complex stands evolves from individual-tree level models that are then aggregated to predict consequences at the stand level^31^. Our developed conditional bivariate probability density function, defined by Eq. 10, outlines the evolution of the tree diameter and height structure via a stand age and can be used to explain some aspects of stand evolution such as the mean values of diameter, height and stem volume and their coefficient of variation, diameter distribution, height distribution, dominant height and the mean stand basal area or stand volume (m^3^/ha).

### Estimating results

The maximum likelihood estimator seeks (see Supplement Method) to make the conditional probability density function the most likely fit to the observed diameter and height estimation dataset $\{({d}_{1}^{i},{h}_{1}^{i}),\,({d}_{2}^{i},{h}_{2}^{i}),\,\ldots ,\,({d}_{{n}_{i}}^{i},{h}_{{n}_{i}}^{i})\}$ at discrete times (ages) $\{{t}_{1}^{i},{t}_{2}^{i},\ldots ,{t}_{{n}_{i}}^{i}\}$ starting at the initial age *t*_0_ = 5, diameter and height *X*(*t*_0_) = *x*_0_ = (0.0, 1.3))^*T*^ from which a given set of SDEs (1) evolves. The parameter estimators, $\mathop{{\theta }^{1}}\limits^{\wedge }=\{{\mathop{\alpha }\limits^{\wedge }}_{d},{\mathop{\alpha }\limits^{\wedge }}_{h},{\mathop{\beta }\limits^{\wedge }}_{d},{\mathop{\beta }\limits^{\wedge }}_{h},{\mathop{\sigma }\limits^{\wedge }}_{11},{\mathop{\sigma }\limits^{\wedge }}_{12},{\mathop{\sigma }\limits^{\wedge }}_{22}\}$ and $\mathop{{\theta }^{2}}\limits^{\wedge }=\{{\mathop{\alpha }\limits^{\wedge }}_{d},{\mathop{\alpha }\limits^{\wedge }}_{h},{\mathop{\beta }\limits^{\wedge }}_{d},{\mathop{\beta }\limits^{\wedge }}_{h},{\mathop{\sigma }\limits^{\wedge }}_{11},{\mathop{\sigma }\limits^{\wedge }}_{12},{\mathop{\sigma }\limits^{\wedge }}_{22},{\mathop{\sigma }\limits^{\wedge }}_{d},{\mathop{\sigma }\limits^{\wedge }}_{h}\}$, were calculated by maximum likelihood and approximated maximum likelihood technique, respectively, realised by Eqs SM2, SM.8 and SM.9 (see Supplement Method), using the NLPSolve procedure in MAPLE 11^32^. The results of the parameter estimates, their standard deviations and the Akaike’s Information Index (AIC)^33^ are summarised in Table 2. The Akaike’s Information Index is defined by:

12 $$AIC=-2\cdot L{L}_{s}(\mathop{{\theta }^{s}}\limits^{\wedge }),\,s=1,2,$$

where *LL*_*s*_(⋅) is defined in the Supplementary Method.

**Table 2:** Estimates of parameters and AIC.

Parameters									AIC
α_d_	β_d_	σ_11_	α_h_	β_h_	σ_22_	σ_12_	σ_d_	σ_h_	
**Univariate fixed effects models**									
35.3599 (56*10^−3^)	0.0280 (1.1*10^−5^)	1.8947 (4.0*10^−4^)	—	—	—	—	—	—	36155
—	—	—	25.1701 (1.8*10^−3^)	0.0406 (1.1*10^−5^)	1.1066 (2.0*10^−4^)	—	—	—	28842
**Univariate mixed effects models**									
36.4617 (3.9*10^−3^)	0.0285 (7.7*10^−6^)	1.4869 (2.6*10^−5^)	—	—	—	—	6.6263 (1.8*10^−3^)	—	35320
—	—	—	26.0732 (1.1*10^−3^)	0.0362 (4.6*10^−6^)	0.5841 (9.3*10^−5^)	—	—	3.9338 (1.0*10^−3^)	25831
**Bivariate fixed effects model**									
35.3462 (1.3*10^−2^)	0.0280 (2.2*10^−5^)	3.5968 (1.0*10^−3^)	25.3301 (4.0*10^−3^)	0.0396 (2.2*10^−5^)	1.1968 (6.1*10^−4^)	3.9187 (9.2*10^−4^)	—	—	62912
**Bivariate mixed effects model**									
35.9030 (9.8*10^−3^)	0.0293 (2.0*10^−5^)	2.3736 (2.2*10^−3^)	26.0039 (2.9*10^−3^)	0.0363 (1.2*10^−5^)	0.3615 (3.0*10^−4^)	0.6334 (6.6*10^−4^)	5.9592 (4.5*10^−3^)	3.6830 (2.6*10^−3^)	58815

The AIC shows that including the random effects into the univariate and bivariate Vasicek-type SDE models improved goodness of fit of the univariate and bivariate conditional probability densities compared with the corresponding fixed effect parameters univariate and bivariate densities.

### Random effects calibration

Calibration means that random effects are predicted using a supplementary dataset of observations taken from a new sampling unit. The theoretical diameter and height trends defined by Eqs 4 and 5 affect the conditional probability density function defined by Eq. 10. Hence, the random effects, ${\varphi }_{d}^{i},{\varphi }_{h}^{i}$, for the *i*th stand, *i* = 1, 2, …, K, for the validation dataset can be calibrated for the diameter by:

13 $$\mathop{{\varphi }_{d}^{i}}\limits^{\wedge }=\frac{1}{{m}_{i}}\sum _{j=1}^{{m}_{i}}\frac{{d}_{j}^{i}-\mathop{{\alpha }_{d}}\limits^{\wedge }+({d}_{0}-\mathop{{\alpha }_{d}}\limits^{\wedge })\cdot \exp (-\mathop{{\beta }_{d}}\limits^{\wedge }\cdot ({t}_{j}^{i}-{t}_{0}))}{1-\exp (-\mathop{{\beta }_{d}}\limits^{\wedge }\cdot ({t}_{j}^{i}-{t}_{0}))},$$

where K is the number of plots in the validation dataset and m_*i*_ is the number of observed trees of the *i*th plot and for the height by:

14 $$\mathop{{\phi }_{h}^{i}}\limits^{\wedge }=\frac{1}{{m}_{i}}\sum _{j=1}^{{m}_{i}}\frac{{h}_{j}^{i}-\mathop{{\alpha }_{h}}\limits^{\wedge }-({h}_{0}-\mathop{{\alpha }_{h}}\limits^{\wedge })\cdot \exp (-\mathop{{\beta }_{h}}\limits^{\wedge }\cdot ({t}_{j}^{i}-{t}_{0}))}{1-\exp (-\mathop{{\beta }_{h}}\limits^{\wedge }\cdot ({t}_{j}^{i}-{t}_{0}))}.$$

### Bivariate diameter and height distribution at a given age

**Figure 1:**
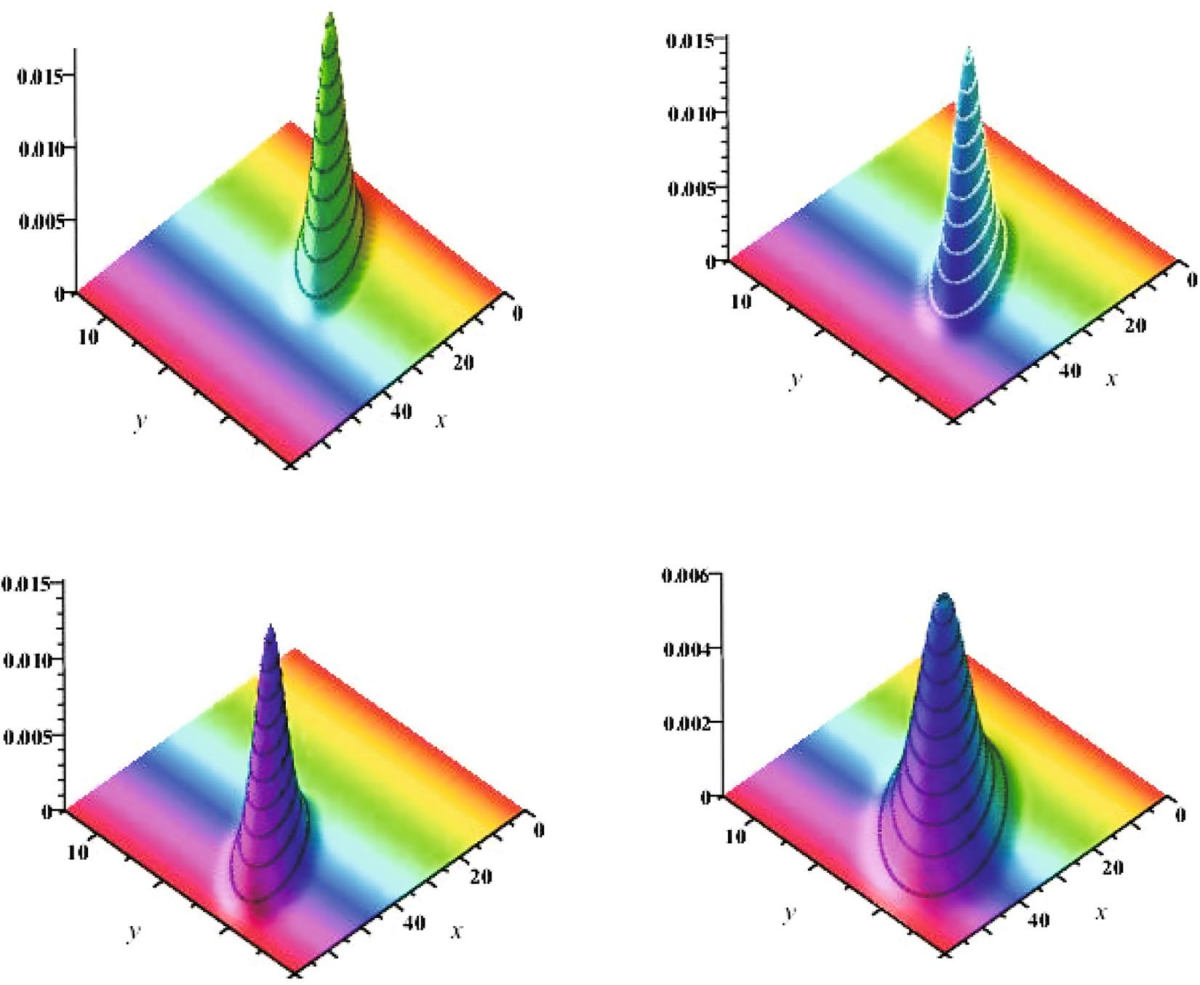
Bivariate estimated probability density functions defined by Eq. 10. First stand (mean of age – 44.0) – top left side; second stand (mean of age – 100.0) – top right side; third stand (mean of age – 145.0) - bottom left side; fixed effects stationary density – bottom right; x – diameter (cm); y – height (m).

The newly developed bivariate conditional probability density function for tree diameter and height is attractive for its simplicity and may be justified from an application perspective. To demonstrate that the data provided by the LNFI (2007–2010) of Scots pine trees do indeed follow the bivariate estimated probability density function defined by Eq. 10 we will use simple graphical techniques to illustrate the goodness of fit. Theoretical validation that a height and diameter dataset observed at discrete ages follows a bivariate probability density function is not easy and there is no simple statistical test. We graphically illustrate goodness of fit of the estimated bivariate density function (Eq. 10) with fixed effect and mixed effect parameters on three randomly selected plots from a validation dataset by plotting the estimated bivariate density functions, their contour plots and the observed datasets. Figure 1 shows the estimated bivariate mixed effect parameters estimated probability density functions for three randomly selected stands from the validation dataset as well as the estimated bivariate fixed effect parameters stationary (time *t* equates to infinity) probability density function (estimates of parameters $\mathop{{\theta }^{1}}\limits^{\wedge }=\{{\mathop{\alpha }\limits^{\wedge }}_{d},{\mathop{\alpha }\limits^{\wedge }}_{h},{\mathop{\beta }\limits^{\wedge }}_{d},{\mathop{\beta }\limits^{\wedge }}_{h},{\mathop{\sigma }\limits^{\wedge }}_{11},{\mathop{\sigma }\limits^{\wedge }}_{12},{\mathop{\sigma }\limits^{\wedge }}_{22}\}$ and $\mathop{{\theta }^{2}}\limits^{\wedge }=\{{\mathop{\alpha }\limits^{\wedge }}_{d},{\mathop{\alpha }\limits^{\wedge }}_{h},{\mathop{\beta }\limits^{\wedge }}_{d},{\mathop{\beta }\limits^{\wedge }}_{h},{\mathop{\sigma }\limits^{\wedge }}_{11},{\mathop{\sigma }\limits^{\wedge }}_{12},{\mathop{\sigma }\limits^{\wedge }}_{22},{\mathop{\sigma }\limits^{\wedge }}_{d},{\mathop{\sigma }\limits^{\wedge }}_{h}\}$ calculated by maximum likelihood procedure presented in the Supplementary Method are summarised in Table 2). The diameter and height random effects ${\varphi }_{d}^{i},{\varphi }_{h}^{i}$ (*i* = 1, 2, 3) for these three randomly selected stands were calibrated by Eqs 13 and 14, respectively.

**Figure 2:**
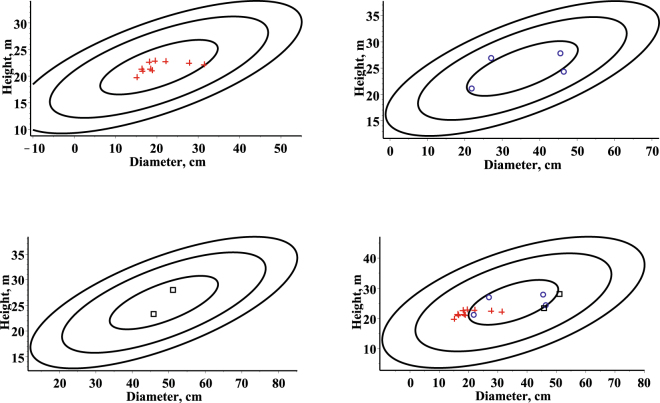
Contour plots (levels: 10^−3^, 10^−6^, 10^−9^) of the estimated bivariate probability density function defined by Eq. 10. First stand (mean of age, diameter and height: 44.0, 20.5, 21.68) – top left side, observed values in red (cross); second stand (mean of age, diameter and height: 100.0, 35.4, 24.9) – top right side, observed values in blue (circle); third stand (mean of age, diameter and height: 145.0, 48.6, 25.6) - bottom left side, observed values in black (box); fixed effects stationary density – bottom right.

The contour plots of the bivariate estimated mixed effects probability density function for three randomly selected stands from the validation dataset and the observed values, together with the contour plot of the bivariate estimated fixed effects stationary (*t* = +infinity) probability density function and the observed values from the same three randomly selected stands from the validation dataset are given in Fig. 2. Figure 2 shows that the mixed effect and fixed effect parameters bivariate estimated probability density functions well capture the main features of the data for three randomly selected stands from the validation dataset. The diameter and height random effects ${\varphi }_{j}^{i},{\varphi }_{j}^{i}$ for these three randomly selected stands were calibrated by Eqs 13 and 14, respectively.

The conditional distribution of the diameter at a given height and age and the conditional distribution of the height at a given diameter and age have univariate normal distributions $N({m}_{d}^{i}(h,t),\,{w}_{d}^{2}(t))$ and$N({m}_{h}^{i}(d,t),$ ${w}_{h}^{2}(t))$, respectively. The mean, variance and coefficient of correlation can be calculated (using Eqs 4–10) in the following forms:

15 $${m}_{d}^{i}(h,t)=E({D}^{i}(t)|{H}^{i}(t)=h)={\mu }_{d}^{i}(t)+\frac{{v}_{d,h}^{2}(t)}{{v}_{h}^{2}(t)}(h-{\mu }_{h}^{i}(t)),$$

16 $${w}_{d}^{2}(t)=Var({D}^{i}(t)|{H}^{i}(t)=h)=(1-r{o}^{2}(t)){\nu }_{d}^{2}(t),$$

17 $$ro(t)=\frac{{\nu }_{d,h}^{2}(t)}{\sqrt{{\nu }_{d}^{2}(t){\nu }_{h}^{2}(t)}},$$

18 $${m}_{h}^{i}(d,t)={\rm E}({H}^{i}(t)|{D}^{i}(t)=d)={\mu }_{h}^{i}(t)+\frac{{\nu }_{d,h}^{2}(t)}{{\nu }_{d}^{2}(t)}(d-{\mu }_{d}^{i}(t)),$$

19 $${w}_{h}^{2}(t)=Var({H}^{i}(t)|{D}^{i}(t)=d)=(1-r{o}^{2}(t)){\nu }_{h}^{2}(t).$$

**Figure 3:**
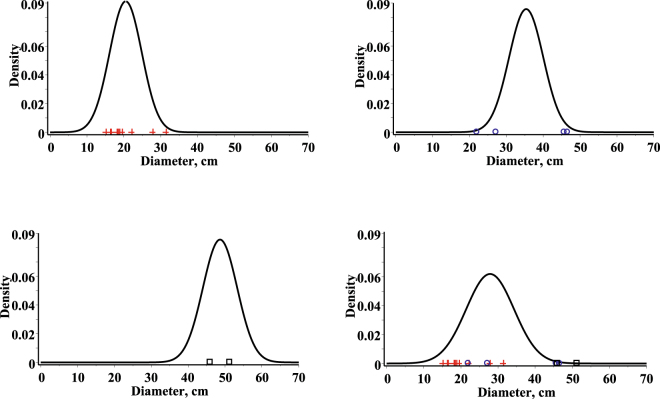
Univariate conditional probability density functions for diameters. First stand (mean of age and height: 44.0, 21.68) – top left side, observed values in red (cross); second stand (mean of age and height: 100.0, 24.9) – top right side, observed values in blue (circle); third stand (mean of age and height: 145.0, 25.6) - bottom left side, observed values in black (box); fixed effects stationary density – bottom right.

**Figure 4:**
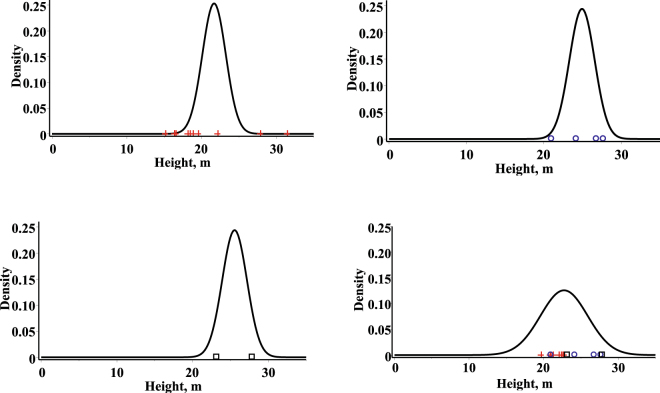
Univariate conditional probability density functions for heights. First stand (mean of age and diameter: 44.0, 20.5) – top left side, observed values in red (cross); second stand (mean of age and diameter: 100.0, 35.4) – top right side, observed values in blue (circle); third stand (mean of age and diameter: 145.0, 48.6) - bottom left side, observed values in black (box); fixed effects stationary density – bottom right.

In summary, Eqs 16 and 19 show us that the univariate conditional distribution of diameter (height) given height (diameter) has an age dependent variance, which is the same for each height (diameter). It is worth mentioning that the result on the height (diameter) independent variance of this study is influenced by properties of bivariate normal distribution. Figures 3 and 4 show the univariate conditional mixed effect parameters probability density functions at a given height and age (diameter and age) for three randomly selected stands from the validation dataset with the observed values, and the univariate conditional fixed effects parameters stationary (*t* = +infinity) probability density function at a given height and age (diameter and age).

### Models of diameter and height

Diameter and height evolution can be formulated using a wide range of mathematical relationships from linearised fixed effect parameters regression equations to nonlinear mixed effect parameters generalised relationships. The mathematical technique for a system of uniform diameter and height regional functions is the approach known as the generalised model. The mixed effects regression models are able to achieve the same results as the generalised model^34^.

Next, we summarise the results concerning the evolution of the diameter or height using univariate and bivariate fixed effects and mixed effects for the Vasicek-type growth models. Recall the models for the diameter and height growth:

univariate fixed effect parameters

20 $${d}_{1}(t)=E(D(t))=E(D(t)|D({t}_{0})={d}_{0})={\alpha }_{d}+({d}_{0}-{\alpha }_{d}){e}^{-{\beta }_{d}(t-{t}_{0})},$$

21 $${h}_{1}(t)=E(H(t))=E(H(t)|H({t}_{0})={h}_{0})={\alpha }_{h}+({h}_{0}-{\alpha }_{h}){e}^{-{\beta }_{h}(t-{t}_{0})},$$

univariate mixed effect parameters

22 $${d}_{2}^{i}(t)=E({D}^{i}(t))=E(D{(t)}^{i}|{D}^{i}({t}_{0})={d}_{0})={\alpha }_{d}+{\varphi }_{d}^{i}-({d}_{0}-({\alpha }_{d}+{\varphi }_{d}^{i})){e}^{-{\beta }_{d}(t-{t}_{0})},$$

23 $${h}_{2}^{i}(t)=E({H}^{i}(t))=E({H}^{i}(t)|{H}^{i}({t}_{0})={h}_{0})={\alpha }_{h}+{\varphi }_{h}^{i}+({h}_{0}-({\alpha }_{h}+{\varphi }_{h}^{i})){e}^{-{\beta }_{h}(t-{t}_{0})},$$

bivariate fixed effect parameters

24 $${d}_{3}(h,t)={\rm{E}}(D(t)|H(t)=h)={d}_{1}(t)+\frac{{v}_{d,h}^{2}(t)}{{v}_{h}^{2}(t)}(h-{h}_{1}(t)),$$

25 $${h}_{3}(d,t)={\rm{E}}(H(t)|D(t)=d)={h}_{1}(t)+\frac{{v}_{d,h}^{2}(t)}{{v}_{d}^{2}(t)}(d-{d}_{1}(t)),$$

bivariate mixed effect parameters

26 $${d}_{4}^{i}(h,t)={\rm{E}}({D}^{i}(t)|{H}^{i}(t)=h)={d}_{2}^{i}(t)+\frac{{v}_{d,h}^{2}(t)}{{v}_{h}^{2}(t)}(h-{h}_{2}^{i}(t)),$$

27 $${h}_{4}^{i}(d,t)={\rm{E}}({H}^{i}(t)|{D}^{i}(t)=d)={h}_{2}^{i}(t)+\frac{{v}_{d,h}^{2}(t)}{{v}_{d}^{2}(t)}(d-{d}_{2}^{i}(t)).$$

Table 3 shows the predictive ability for all newly developed fixed- and mixed effect parameters diameter and height models for both estimation and validation datasets (using the estimates of parameters $\mathop{{\theta }^{1}}\limits^{\wedge }=\{{\mathop{\alpha }\limits^{\wedge }}_{d},{\mathop{\alpha }\limits^{\wedge }}_{h},{\mathop{\beta }\limits^{\wedge }}_{d},$ ${\mathop{\beta }\limits^{\wedge }}_{h},{\mathop{\sigma }\limits^{\wedge }}_{11},{\mathop{\sigma }\limits^{\wedge }}_{12},{\mathop{\sigma }\limits^{\wedge }}_{22}\}$ and $\mathop{{\theta }^{2}}\limits^{\wedge }=\{{\mathop{\alpha }\limits^{\wedge }}_{d},{\mathop{\alpha }\limits^{\wedge }}_{h},{\mathop{\beta }\limits^{\wedge }}_{d},{\mathop{\beta }\limits^{\wedge }}_{h},{\mathop{\sigma }\limits^{\wedge }}_{11},{\mathop{\sigma }\limits^{\wedge }}_{12},{\mathop{\sigma }\limits^{\wedge }}_{22},{\mathop{\sigma }\limits^{\wedge }}_{d},{\mathop{\sigma }\limits^{\wedge }}_{h}\}$ presented in Table 2, and the random effects for diameter and height calibrated by Eqs 13 and 14, respectively). The estimated diameter evolution defined by Eq. 26 and the estimated height evolution defined by Eq. 27 were the best functions, with an increase in the coefficient of determination (R^2^) and decreases in the absolute prediction bias (AB) and the error (AE) for both estimation and validation datasets.

**Table 3:** Statistical indexes for all models applied to the estimation and validation datasets.

Model	Estimation				Validation			
	B, m (PB, %)	AB, m (PAB, %)	AE, m	R^2^	B, m (PB, %)	AB, m (PAB, %)	AE, m	R^2^
**Diameter**								
1	0.0346 (−7.1912)	6.0611 (23.6162)	7.7593	0.1915	−0.1944 (−8.3678)	6.1214 (23.9642)	7.8347	0.2136
2	0.0147 (−4.5528)	4.2463 (16.4927)	5.4387	0.6027	−0.0025 (−3.7089)	4.0124 (15.5738)	5.2636	0.6447
3	**0.0186** (−4.5137)	4.8389 (18.3712)	6.3144	0.4642	−0.1555 (−5.2887)	4.8765 (18.4629)	6.3604	0.4809
4	0.0212 **(**−**2.3160)**	**3.0770 (11.6959)**	**3.9890**	**0.7863**	−**0.0009 (**−**1.8057)**	**3.0381 (11.5048)**	**4.0440**	**0.7903**
**Height**								
1	0.0070 (−3.5060)	3.0014 (14.9750)	3.8506	0.2405	−0.0359 (−3.7091)	3.0078 (14.8898)	3.8385	0.2491
2	0.0018 (−1.0817)	1.4248 (6.9093)	1.8698	0.8209	−0.0004 (−0.8606)	1.4514 (6.8880)	1.9351	0.8091
3	−**0.0005** (−2.5673)	2.4610 (12.3000)	3.1442	0.4932	0.0177 (−2.4110)	2.4405 (12.0220)	3.1192	0.5034
4	−0.0060 **(**−**0.6738)**	**1.0835 (5.2324)**	**1.3934**	**0.9005**	6.2*10 ^−6^ **(**−**0.5133)**	**1.1291 (5.3267)**	**1.4832**	**0.8876**

The best values of the statistical indexes are in bold, the mean prediction bias $(B=\frac{1}{n}\sum _{i=1}^{n}({y}_{i}-\mathop{{y}_{i}}\limits^{\wedge }))$ and the percentage mean prediction bias $(PB=\frac{1}{n}\sum _{i=1}^{n}\frac{{y}_{i}-\mathop{{y}_{i}}\limits^{\wedge }}{{y}_{i}}\cdot 100)$, the absolute mean prediction bias $(AB=\frac{1}{n}\sum _{i=1}^{n}|{y}_{i}-\mathop{{y}_{i}}\limits^{\wedge }|)$ and the percentage mean absolute prediction bias $(AB=\frac{1}{n}\sum _{i=1}^{n}|\frac{{y}_{i}-\mathop{{y}_{i}}\limits^{\wedge }}{{y}_{i}}|)$, an adjusted root mean square error $(AE=\sqrt{{B}^{2}+\frac{1}{n-1}\sum _{i=1}^{n}{({y}_{i}-\mathop{{y}_{i}}\limits^{\wedge }-B)}^{2}})$ and an adjusted coefficient of determination $({R}^{2}=1-\frac{n-1}{n-p}\frac{\sum _{i=1}^{n}{({y}_{i}-\mathop{{y}_{i}}\limits^{\wedge })}^{2}}{\sum _{i=1}^{n}{({y}_{i}-\overline{y})}^{2}})$. Here p is the number of parameters in the model (for fixed effects scenario p = 7 and for mixed effects scenario p = 9), $n=\sum _{i=1}^{M}{n}_{i}$ is the total number of observations used to fit the model, M is the number of stands, *n*_*i*_ is the number of measured trees *i*n the *i*th stand, *y*_*i*_, $\mathop{{y}_{i}}\limits^{\wedge }$ and $\overline{y}$ are the measured, estimated and average values of the dependent variable (diameter or height).

**Figure 5:**
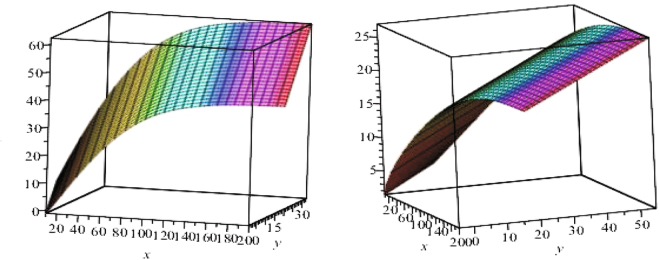
Estimated evolution of diameter and height for randomly selected stand (mean of age, diameter and height: 44.0, 20.5, 21.68). Left – diameter versus height and age (x – age, y – height); right – height versus diameter and age (x – age, y – diameter).

The estimated evolution of the diameter, $d={d}_{4}^{i}(h,t)$, subject to the height and age, and the estimated evolution of the height, $h={h}_{4}^{i}(d,t)$, subject to the diameter and age, for a randomly selected stand from the validation dataset are shown in Fig. 5, where we have assumed that the values of the predictor variable height (diameter) lie within a band around the mean ${\mu }_{h}^{i}(t)$ $({\mu }_{d}^{i}(t))$ with a width of three standard deviations $\sqrt{{\nu }_{h}^{2}(t)}$ $(\sqrt{{\nu }_{d}^{2}(t)})$.

In Supplementary Fig. S1, the residuals of the diameter models defined by Eqs 20, 22, 24 and 26, and the LOWESS line (Locally Weighted Scatterplot Smoothing line) are plotted against the predicted diameter values. In Supplementary Fig. S2, the residuals of the height models defined by Eqs 21, 23, 25 and 27, and the LOWESS line are plotted against the predicted height values. Supplementary Figs S1 and S2 show that the residuals that were calculated using the bivariate mixed effects scenario are distributed more symmetrically around zero, with approximately constant variance, compared with the other scenarios. Therefore, the bivariate models incorporating the random effects of the stands were the best models for predicting diameter and height growth of individual Scots pine trees in the study area (see Supplementary Figs S1 and S2). A non-parametric smoothing line, called a LOWESS line, shows a clear trend of predicted height; however, at the predicted diameter extremes the deviations are dictated by relatively little data.

The derived univariate conditional distribution of a diameter at a given height and age, and the derived univariate conditional distribution of a height at a given diameter and age, have normal distributions $N({m}_{d}^{i}(h,t),\,{w}_{d}^{2}(t))$ and $N({m}_{h}^{i}(d,t),\,{w}_{h}^{2}(t))$, respectively, and describe the knowledge of the predicted mean and uncertainty about the mean completely. These distributions can be used to describe the accuracy of predicted values by calculating the confidence intervals.

### Stem structure

**Figure 6:**
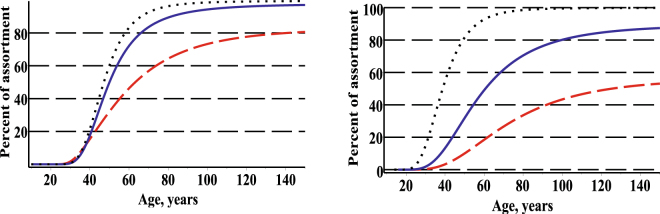
Estimated evolution of percentage of trees for three randomly selected stands and two scenarios. First stand – red on dash (mean of age, diameter and height: 44.0, 20.5, 21.68); second stand – blue on solid (mean of age, diameter and height: 100.0, 35.4, 24.9); third stand – black on dot (mean of age, diameter and height: 145.0, 48.6, 25.6); left scenario – diameter > 25, height > 20; right scenario – diameter > 30.

The bivariate conditional probability density function defined by Eq. 10 allows us to find the evolution of the percentage of trees in a particular stand that achieve fixed sizes for the diameter and height. Figure 6 shows the evolution of the percentage of two assortments for three randomly selected stands from the validation dataset.

### Slenderness ratio

**Figure 7:**
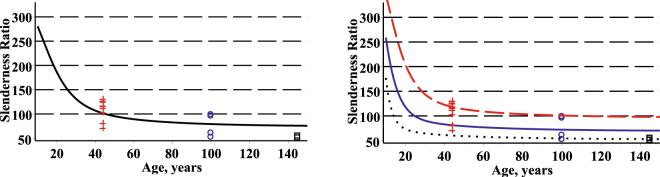
Estimated evolution of mean slenderness ratio for three randomly selected stands. Left –fixed effects scenario; right – mixed effects scenario; first stand – red on dash (mean of age, diameter and height: 44.0, 20.5, 21.68), observed values in cross); second stand – blue on solid (mean of age, diameter and height: 100.0, 35.4, 24.9), observed values in circle; third stand – black on dot (mean of age, diameter and height: 145.0, 48.6, 25.6), observed values in box.

The slenderness coefficient is an important characteristic for indexing tree resistance to wind throw and snow damage. Figure 7 shows the evolution of the mean slenderness coefficient for three randomly selected plots. The slenderness of trees decreases with increasing stand age. For the bivariate fixed effect and mixed effect SDEs height and diameter distribution models the evolution of the slenderness is defined as follows:

28 $$SR(t)={\int }_{-\infty }^{+\infty }{\int }_{-\infty }^{+\infty }\frac{h}{d}\cdot f(d,h,t|\mathop{\theta }\limits^{\wedge })\cdot dd\cdot dh,\,\mathop{\theta }\limits^{\wedge }\in \{(\mathop{{\theta }^{1}}\limits^{\wedge },0,0),(\mathop{{\theta }^{2}}\limits^{\wedge },\mathop{{\varphi }_{d}}\limits^{\wedge },\mathop{{\varphi }_{h}}\limits^{\wedge })\},$$

### Mean and coefficient of variation of stem volume

For forestry applications, the most commonly used approach is to derive measures to estimate forest variables such as mean tree diameter, height and stem volume.

First, the bivariate fixed effects and mixed effects conditional probability density function of the diameter and height allows us to calculate the evolution of the mean stem volume in the following form:

29 $$\overline{V}(t)={\int }_{-\infty }^{+\infty }{\int }_{-\infty }^{+\infty }V(d,h)\cdot f(d,h,t|\mathop{\theta }\limits^{\wedge })\cdot dd\cdot dh,\,\mathop{\theta }\limits^{\wedge }\in \{(\mathop{{\theta }^{1}}\limits^{\wedge },0,0),(\mathop{{\theta }^{2}}\limits^{\wedge },\mathop{{\varphi }_{d}}\limits^{\wedge },\mathop{{\varphi }_{h}}\limits^{\wedge })\},$$

where *V*(*d*, *h*) is the individual stem volume regression function of power form^35^:

30 $$V(d,\,h)={\beta }_{1}{d}^{{\beta }_{2}}{h}^{{\beta }_{3}}.$$

**Figure 8:**
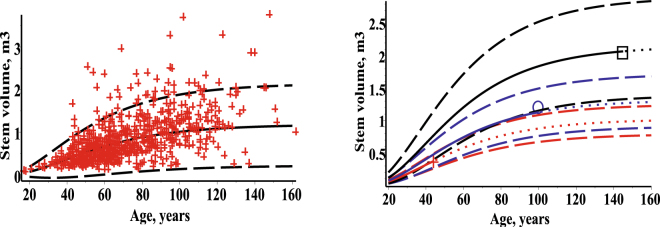
Estimated evolution of mean stem volume and mean ± 1.65*standard deviation (confidence bands). Left – fixed effect scenario: mean stem volume trend – solid; confidence bands – dash; observed mean stem volumes in cross. Right – mixed effect scenario for three randomly selected stands: first stand – red (mean of age, diameter and height: 44.0, 20.5, 21.68) and observed mean stem volume – cross; second stand – blue (mean of age, diameter and height: 100.0, 35.4, 24.9) and observed mean stem volume – circle; third stand – black (mean of age, diameter and height: 145.0, 48.6, 25.6) and observed mean stem volume – box. Mean stem volume trend from initial age to observed stand age – solid and from this age forward (forecast) – dot. Confidence bands – dash.

Estimates of the parameters β_1_ β_2_, and β_3_ were calculated by a weighted least squares technique^36^. The estimators and their standard deviations (in parenthesis) are, ${\mathop{\beta }\limits^{\wedge }}_{1}={\rm{5.8}}\ast {{\rm{10}}}^{-{\rm{5}}}\,(5{\rm{.8}}\ast {{\rm{10}}}^{-{\rm{6}}})$, ${\mathop{\beta }\limits^{\wedge }}_{2}={\rm{1.8801}}\,(0\mathrm{.028})$, ${\mathop{\beta }\limits^{\wedge }}_{3}={\rm{0.9723}}\,(0\mathrm{.045})$. The estimated evolution of the mean stem volume in a stand for the fixed effect and mixed effect models and $\pm 1.65\cdot S{D}_{\overline{V}}(t)$ confidence bands (if we assume normality then they covers 90%) are shown in Fig. 8 for all stands (fixed effect scenario) and three randomly selected stands (mixed effect scenario) from the validation dataset, respectively. Observed values of mean stem volumes in all stands were defined by the average of stem volumes calculated by the regression (Eq. 30). For the mixed effect scenario the mean stem volume estimate proves satisfactory, with the mean prediction bias (the percentage mean prediction bias) −0.0158 m^3^ (−3.28%) and with the mean prediction absolute bias (the percentage mean prediction absolute bias) 0.0322 m^3^ (5.07%). For the validation dataset the percent of the mean stem volume variation explained attains high levels too, 99.51%, for the mixed effect methodology.

Second, the bivariate fixed effects and mixed effects conditional probability density functions of the diameter and height allow us to estimate stem volume variability by the coefficient of variation. We estimate the evolution of the standard deviation of the stem volume in the following form:

31 $$S{D}_{V}(t)=\sqrt{{\int }_{-\infty }^{+\infty }{\int }_{-\infty }^{+\infty }V{(d,h)}^{2}\cdot f(d,h,t|\mathop{\theta }\limits^{\wedge })\cdot dd\cdot dh-\overline{V}{(t)}^{2}},\,\mathop{\theta }\limits^{\wedge }\in \{(\mathop{{\theta }^{1}}\limits^{\wedge },0,0),(\mathop{{\theta }^{2}}\limits^{\wedge },\mathop{{\varphi }_{d}}\limits^{\wedge },\mathop{{\varphi }_{h}}\limits^{\wedge })\}.$$

Hence, the coefficient of variation takes the following form:

32 $$CV(t)=\frac{S{D}_{V}(t)}{\overline{V}(t)}\cdot 100.$$

**Figure 9:**
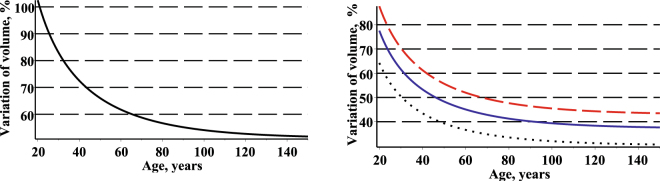
Coefficient of variation of stem volume for three randomly selected stands. Left –fixed effects scenario; right – mixed effects scenario; first stand – red on dash (mean of age, diameter and height: 44.0, 20.5, 21.68); second plot – blue on solid (mean of age, diameter and height: 100.0, 35.4, 24.9); third stand – black on dot (mean of age, diameter and height: 145.0, 48.6, 25.6).

The coefficient of variation of stem volume defined by Eq. 32 is a statistical measure of the dispersion of the volumes calculated by Eq. 30 data points around the mean stem volume calculated by Eq. 29. The coefficient of variation of stem volume is a useful statistic for comparing the degree of variation of stem volume from one stand to another, even if the means are drastically different from one another. Figure 9 shows a plot of the coefficient of variation as a function of stand age using the mean trend and standard deviation functions of stem volume. The coefficient of variation of the stem volume evolves into a stationary coefficient of variation. The coefficient of variation based on stem volume decreases with an increase in stand age.

### Stand volume per ha

**Figure 10:**
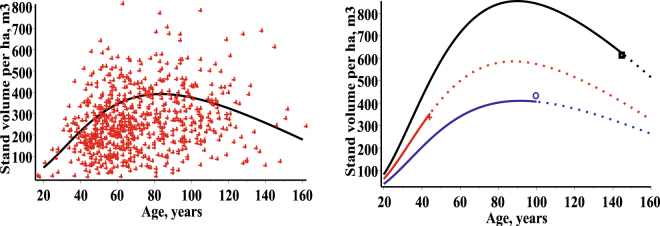
Estimated evolution of stand volume per ha (m^3^) using fixed and mixed effect scenarios. Left – fixed effect scenario: stand volume trend – solid and observed stand volumes – crosses. Right – mixed effect scenario for three randomly selected stands: first stand – red (mean of age, diameter and height: 44.0, 20.5, 21.68) and observed stand volume – cross; second stand – blue (mean of age, diameter and height: 100.0, 35.4, 24.9) and observed volume – circle; third stand – black (mean of age, diameter and height: 145.0, 48.6, 25.6) and observed stand volume – box. Stand volume trend from initial age to observed stand age – solid and from observed age forward (forecast) – dot.

Estimation of stand volume per ha is important for sustainable forest management. Methods to forecast stand volume per ha (or stem volume structure) for a short or long time period are necessary to ensure forest management schemes. Traditionally, estimating stand volume per ha includes several steps, such as the choice of stem volume regression model, the choice of a height-diameter model when tree height data are incomplete, the expansion of a sample volume to the number of trees it represents per unit area or the choice of stand volume per ha model developed by replacing the original tree variables into equivalent stand variables^37^ (tree height are replaced by dominant height and diameter at breast height by quadratic mean diameter). The SDEs framework enables us to characterise a stand volume per ha as a function of any specified stand age, *t*, in the following form:

33 $${V}_{S}(t)={m}_{N}(t)\cdot {\int }_{-\infty }^{+\infty }{\int }_{-\infty }^{+\infty }V(d,h)\cdot f(d,h,t|\mathop{\theta }\limits^{\wedge })\cdot dd\cdot dh,\,\mathop{\theta }\limits^{\wedge }\in \{(\mathop{{\theta }^{1}}\limits^{\wedge },0,0),(\mathop{{\theta }^{2}}\limits^{\wedge },\mathop{{\varphi }_{d}}\limits^{\wedge },\mathop{{\varphi }_{h}}\limits^{\wedge })\},$$

here *m*_*N*_(*t*) is the number of trees per ha at a given age, *t*. The Vasicek-type univariate SDE that describes the development of the number of trees per ha, *N* ^*i*^(*t*), is defined by $d{N}^{i}(t)=\beta (\alpha +{\varphi }_{N}^{i}-{N}^{i}(t))\cdot dt+\sigma \cdot {W}^{i}(t)$, *i* = 1, 2, …, M, *P*(*N* ^*i*^(200) = *δ*) = 1, here *δ* is unknown parameter representing the number of trees at the age of the 200 years, ${\varphi }_{N}^{i}$, is normally distributed random variable with zero mean and constant variance, ${\varphi }_{N}^{i} \sim N(0;\,{\sigma }_{N}^{2})$. The conditional distribution of the number of trees per ha at a given age has a univariate normal distribution $N({\mu }_{N}^{i}(t);\,{w}_{N}^{2}(t))$, with mean ${\mu }_{N}^{i}(t)=\alpha +{\varphi }_{N}^{i}-(\delta -(\alpha +{\varphi }_{N}^{i})\cdot {e}^{\beta (t-200)}$ and variance ${w}_{N}^{2}(t)=\frac{{\sigma }^{2}(1-{e}^{2\beta (t-200)})}{2\beta }$. The estimates of the parameters and their standard deviations (in parenthesis) are: for the fixed effects scenario $\hat{\alpha }$ = 41215.9217 (74.6637), $\hat{{\beta }}$ = 8.84 * 10^−5^ (1.62 * 10^−7^), $\hat{\sigma }$ = 18.9385 (0.0029), $\hat{{\delta }}$ = 10.0 (0.1744); for the mixed effects scenario $\hat{\alpha }$ = 2547.4181 (0.4148), $\hat{{\beta }}$ = 0.0012 (8.86 * 10^−8^), $\hat{\sigma }$ = 5.8849 (0.0015), $\hat{{\delta }}$ = 94.7886 (0.0540), ${\mathop{\sigma }\limits^{\wedge }}_{N}=1516.9969\,(0\mathrm{.4449})$. Figure 10 shows the evolution of the stand volume per ha as a function of a stand age using the fixed effects scenario and the mixed effects scenario (for three randomly selected stands from the validation dataset).

## Conclusions

We focus on discussion of the bivariate Vasicek-type stochastic process that drives the density evolution of the tree diameter and height structure via stand age. In this study, principal concepts of the bivariate mixed effect parameters Vasicek-type SDE in simple terms are presented and results of the diameter and height distribution, mean diameter, mean height, mean stem volume and stand volume per ha evolution via stand age are illustrated using the Lithuanian National Forest dataset from 2007 to 2010 for Scots pine trees. A new SDE framework for bivariate diameter and height distribution modelling is demonstrated using stand-level random effects. The newly developed conditional probability density functions are easy applied for the modelling of the evolution of the stand attributes via stand age.

The newly developed bivariate conditional probability density functions allowed us to calculate the evolution, in the forward and backward directions, of the mean diameter, height, dominant height, stem volume, stem volume of a stand and the uncertainties in these characteristics completely for a given stand age. The derived conditional probability density can be used to describe the accuracy of the predicted values (diameter, height and stem volume) in the sense of confidence bands.

It is easy to estimate fixed and random effects of the bivariate SDE using large datasets, which in this case was provided by the National Forest Inventories (one cycle). Moreover, the newly developed bivariate mixed effects conditional probability density function is an appropriate model for examination of diameter and height distributions of uneven-aged forest stands.

The present paper attempts to expand the scientific knowledge related to the evolution of tree diameter and height distributions in a forest stand by using SDEs, random effect technique and their possible applications to the modelling of the mean diameter, height, stem volume and stand volume per ha of a stand. Although this study has some limitations (e.g. estimation and validation datasets are not provided with remeasurement cycles of plots), these findings contribute to a better understanding of how stand growth and yield attributes be related to the stand age. Therefore, in our opinion, this study represents one of the first attempts to examine the evolution of growing stock resources, their structure and changes using the bivariate random process methodology and utilizing the data from one cycle of the LNFI. For a better understanding of the tree number and stand volume per ha dynamics remeasurement data from permanent plots (LNFI) could be utilized.

The trivariate (diameter, height and number of trees per ha) distributional model would be superior to the bivariate (diameter and height) distributional model in accordance to the underlying covariance structure driving changes in the tree diameter, height and number of trees per ha.

The number of trees per ha plays a central role for stand growth, but it’s representation in the LNFI dataset is limited by data availability. Missing data on initial density of stand (number of trees per ha at the age of t_0_ = 5), restricted ability to develop a trivariate model.

Further research on the interplay between tree diameter, height and number of trees per ha using trivariate diffusion process and remeasurement data (2 or 3 cycles) from the LNFI would improve the consequences for the tree number and stand volume per ha evolution.

The analysis of the mixed effect SDE bivariate model by the specification of one random effect structure that used estimation based on a maximum likelihood procedure is not properly supported by the LNFI dataset. On the other hand, the model with the structure of all possible random effect components included may not converge.

## Electronic supplementary material

Supplementary Information 1

Supplementary Information 2
