# Supplementary material for: A new paradigm in modelling the evolution of a stand via the distribution of tree sizes
Source: Sci Rep. 2017 Nov 20;7:15875. doi: 10.1038/s41598-017-16100-2 (PMC5696519; doi:10.1038/s41598-017-16100-2)
Supplement: Supplementary file 1 — Supplementary Information 1 [file 41598_2017_16100_MOESM1_ESM.doc]

**A new paradigm in modelling the evolution of a stand via the distribution of tree sizes**

Petras Rupšys^1,2,*^ & Edmundas Petrauskas^1^

^1^Institute of Forest Management and Wood Sciences, Aleksandras Stulginskis University, Studentu 11, Kaunas, LT-53361, Lithuania. ^2^Centre of Mathematics, Physics and Information Technologies, Aleksandras Stulginskis University, Studentu 11, Kaunas, LT-53361, Lithuania. Correspondence and requests for materials should be addressed to P.R. (email: petras.rupsys@asu.lt)

**Supplementary Method** Approximated maximum likelihood procedure

The SDE model defined by Eq. 1 can be fitted to diameter and height sample $\left\{\left(d_{1}^{i},h_{1}^{i}\right), \left(d_{2}^{i},h_{2}^{i}\right), \ldots, \left(d_{n_{i}}^{i},h_{n_{i}}^{i}\right)\right\}$ at discrete times (ages) $\left\{t_{1}^{i},t_{2}^{i},\ldots,t_{n_{i}}^{i}\right\}$ (*n_i_* is the number of observed trees of the i*th* plot, i=1, 2, …, M) by maximum likelihood procedure. In the sequel, $\theta^{1}=\left\{\alpha_{d},\alpha_{h},\beta_{d},\beta_{h},\sigma_{11},\sigma_{12},\sigma_{22}\right\}$ and $\theta^{2}=\left\{\alpha_{d},\alpha_{h},\beta_{d},\beta_{h},\sigma_{11},\sigma_{12},\sigma_{22},\sigma_{d},\sigma_{h}\right\}$. The associated maximum likelihood function for the bivariate fixed effects parameters SDE (diameter and height) model (in this case the parameters of random effects $\varphi_{d}^{i}$ and $\varphi_{h}^{i}$ are assumed to be equal to the mean value $E\left(\varphi_{d}^{i}\right)=0$ and $E\left(\varphi_{h}^{i}\right)=0$, respectively, i=1, 2, …, M), takes the following form:

$L_{1}(\theta^{i})=\prod_{i=1}^{M} \prod_{j=1}^{n_{i}} f\left(d_{j}^{i},h_{j}^{i},t_{j}^{i}\left|\theta^{1},0,0\right.\right)$, (SM.1)

and the maximum log-likelihood function as:

$LL_{1}(\theta^{1})=\sum_{i=1}^{M} \sum_{j=1}^{n_{i}} \ln\left(f\left(d_{j}^{i},h_{j}^{i},t_{j}^{i}\left|\theta^{1},0,0\right.\right)\right)$, (SM.2)

where *n_i_* is the number of observed trees of the i*th* plot $i=1, 2, ..., M$, $\theta^{1}$ are the fixed effects parameters (the same for all plots), conditional probability density function $f\left(d_{j}^{i},h_{j}^{i},t_{j}^{i}\left|\theta^{1},0,0\right.\right)$ takes the form defined by Eq. 10.

The maximum likelihood function for the bivariate mixed effects SDE (diameter and height) model takes the following form:

$L_{2}(\theta^{2})=\prod_{i=1}^{M} \prod_{j=1}^{n_{i}} \int_{-\infty}^{+\infty} \int_{-\infty}^{+\infty} f\left(d_{j}^{i},h_{j}^{i},t_{j}^{i}\left|\theta^{1},\varphi_{d}^{i},\varphi_{h}^{i}\right.\right)\cdot p(\varphi_{d}^{i}\left|\sigma_{d}\right.)\cdot p(\varphi_{h}^{i}\left|\sigma_{h}\right.)\cdot d\varphi_{d}^{i}\cdot d\varphi_{h}^{i}$, (SM.3)

and the maximum log-likelihood function is:

$LL_{2}(\theta^{2})=\sum_{i=1}^{M} \int_{-\infty}^{+\infty} \int_{-\infty}^{+\infty} \left(\sum_{j=1}^{n_{i}} \ln\left(f\left(d_{j}^{i},h_{j}^{i},t_{j}^{i}\left|\theta^{1},\varphi_{d}^{i},\varphi_{h}^{i}\right.\right)\right)+\ln\left(p(\varphi_{d}^{i}\left|\sigma_{d}\right.)\right)+\ln\left(p(\varphi_{h}^{i}\left|\sigma_{h}\right.)\right)\right)\cdot d\varphi_{d}^{i}\cdot d\varphi_{h}^{i}$, (SM.4)

where $\theta^{2}$ are fixed effects parameters (the same for all plots), $\varphi_{d}^{i}$ and $\varphi_{h}^{i}$ are independent random effects (plot specific), both follow normal distributions with *0* mean and constant variances $\sigma_{d}^{2}$ and $\sigma_{h}^{2}$, respectively, and the normal density functions of the random effects are $p\left(\varphi_{d}^{i}\left|\sigma_{d}\right.\right)$ and $p\left(\varphi_{h}^{i}\left|\sigma_{h}\right.\right)$, respectively.

Because the integral in Eq. SM.3 does not have a closed form solution and analytic expression is known, we will use the Laplace method^S1^. Let us define a vector $\varphi^{i}=\left(\varphi_{d}^{i}, \varphi_{h}^{i}\right)$ and a function $g:R\to R$ as follows^S2^:

$g\left(\varphi^{i}\left|\theta^{2}\right.\right)=\sum_{j=1}^{n_{i}} \ln\left(f\left(d_{j}^{i},h_{j}^{i},t_{j}^{i}\left|\theta^{1},\varphi_{d}^{i},\varphi_{h}^{i}\right.\right)\right)+\ln\left(p(\varphi_{d}^{i}\left|\sigma_{d}\right.)\right)+\ln\left(p(\varphi_{h}^{i}\left|\sigma_{h}\right.)\right)$, $i=1, 2, ..., M$. (SM.5)

The Laplace approximation to $\int_{-\infty}^{+\infty} \int_{-\infty}^{+\infty} e^{g_{K}\left(\varphi^{i}\left|\theta^{2}\right.\right)}\cdot d\varphi_{d}^{i}\cdot d\varphi_{h}^{i}$, $i=1, 2, ..., M$, is based on a second-order Taylor series expansion about mode $\mathop{\varphi^{i}}\limits^{\wedge} =\left(\mathop{\varphi_{d}^{i}}\limits^{\wedge} ,\mathop{\varphi_{h}^{i}}\limits^{\wedge} \right)$, $i=1, 2, ..., M$:

$\begin{matrix} \int_{-\infty}^{+\infty} \int_{-\infty}^{+\infty} e^{g_{K}\left(\varphi^{i}\left|\theta^{2}\right.\right)}\cdot d\varphi_{d}^{i}\cdot d\varphi_{h}^{i} \\ \approx e^{g_{K}\left(\mathop{\varphi^{i}}\limits^{\wedge} \left|\theta^{2}\right.\right)}\int_{-\infty}^{+\infty} \int_{-\infty}^{+\infty} \exp\left(\frac{1}{2}\left(\varphi^{i}-\mathop{\varphi^{i}}\limits^{\wedge} \right)^{T}\frac{\partial^{2}g_{K}\left(\mathop{\varphi^{i}}\limits^{\wedge} \left|\theta^{2}\right.\right)}{\partial\varphi_{d}^{i}\partial\varphi_{h}^{i}}\left(\varphi^{i}-\mathop{\varphi^{i}}\limits^{\wedge} \right)\right)\cdot d\varphi_{d}^{i}\cdot d\varphi_{h}^{i} \end{matrix}$, (SM.6)

where $\mathop{\varphi^{i}}\limits^{\wedge}$ is the global max of $g\left(\cdot\left|\theta^{2}\right.\right)$. Then, the Laplace approximation of $\ln\left(\int_{-\infty}^{+\infty} \int_{-\infty}^{+\infty} e^{g\left(\varphi_{i}\left|\theta^{2}\right.\right)}\cdot d\varphi_{d}^{i}\cdot d\varphi_{h}^{i}\right)$ takes the following form, $i=1, 2, ..., M$:

$\ln\left(\int_{-\infty}^{+\infty} \int_{-\infty}^{+\infty} e^{g\left(\varphi^{i}\left|\theta^{2}\right.\right)}\cdot d\varphi_{d}^{i}\cdot d\varphi_{h}^{i}\right)\approx g\left(\mathop{\varphi^{i}}\limits^{\wedge} \left|\theta^{2}\right.\right)+\ln\left(2\pi\right)-\frac{1}{2}\ln\left(\det\left(\left[-\frac{\partial^{2}g\left(\varphi^{i}\left|\theta^{2}\right.\right)}{\partial\varphi_{d}^{i}\partial\varphi_{h}^{i}}\right]\right)_{\varphi^{i}=\mathop{\varphi^{i}}\limits^{\wedge} }\right)$, (SM.7)

where:

$\mathop{\varphi^{i}}\limits^{\wedge} =\mathop{\mathrm{argmax}}\limits_{\varphi^{i}} g\left(\varphi^{i}\left|\theta^{2}\right.\right)$, $i=1, 2, ..., M$. (SM.8)

The maximum log-likelihood function for the bivariate mixed-effects SDE (diameter and height) model is approximately given by:

$LL_{2}(\theta^{2})\approx\sum_{i=1}^{M} \left(g\left(\mathop{\varphi^{i}}\limits^{\wedge} \left|\theta^{2}\right.\right)+\ln\left(2\pi\right)-\frac{1}{2}\ln\left(\det\left(\left[-\frac{\partial^{2}g\left(\varphi^{i}\left|\theta^{2}\right.\right)}{\partial\varphi_{d}^{i}\partial\varphi_{h}^{i}}\right]\right)_{\varphi^{i}=\mathop{\varphi^{i}}\limits^{\wedge} }\right)\right)$. (SM.9)

The maximization of $LL_{2}\left(\theta^{2}\right)$ is a two-step optimization problem. The internal optimization step estimates the vector $\mathop{\varphi^{i}}\limits^{\wedge}$ for ever*y* plot $i=1, 2, ..., M$ with Eq. SM.8. The external optimization step maximizes $LL_{2}\left(\theta^{2}\right)$ after plugging the $\mathop{\varphi^{i}}\limits^{\wedge}$ into Eq. SM.9. These two steps are iterated until convergence.

To assess the asymptotic standard errors of the maximum likelihood estimators the Fisher^S3^ information matrix was used. The approximate asymptotic variance of the approximated maximum likelihood estimators (Eq. SM.9) was calculated by the inverse of observed Fisher information matrix. By defining the vector $\left(LL_{s}(\theta^{k})\right)^{'}\equiv\frac{\partial LL_{s}(\theta^{s})}{\partial\theta_{i}^{s}}$, and the matrix $\left(LL_{s}(\theta^{s})\right)^{''}\equiv\left[\frac{\partial^{2}LL_{s}(\theta^{s})}{\partial\theta_{i}^{s}\partial\theta_{j}^{s}}\right]^{T}$, s=1,2, the observed Fisher information matrix takes the following form:

$\mathop{I}\limits^{\sim} (\mathop{\theta^{s}}\limits^{\wedge} )=\left[-\frac{\partial^{2}LL_{s}(\theta^{s})}{\partial\theta_{i}^{s}\partial\theta_{j}^{s}}\right]^{T}\left|\theta^{s}=\mathop{\theta^{s}}\limits^{\wedge} \right.$, s=1,2. (SM.10)

The approximate asymptotic standard errors of the fixed effects parameters are defined by the diagonal elements of the matrix $\left[\mathop{I}\limits^{\sim} (\mathop{\theta^{s}}\limits^{\wedge} )\right]^{-1}$, s=1,2:

$SE\left(\mathop{\theta^{s}}\limits^{\wedge} \right)=\sqrt{\left[\mathop{I}\limits^{\sim} _{ii}(\mathop{\theta^{s}}\limits^{\wedge} )\right]^{-1}*\frac{1}{n}}$ (SM.11)

**References**

S1. Joe, H. Accuracy of Laplace approximation for discrete response mixed models. *Comput. Stat. Data An*. **52**, 5066–5074 (2008).

S2. Picchini, U., Ditlevsen, S. & De Gaetano, A. Practical estimation of high dimensional stochastic differential mixed-effects models. *Comput. Stat. Data An.* **55(3)**, 1426-1444 (2011).

S3. Fisher, R. A. On the mathematical foundations of theoretical statistics. *Philos. T. Roy. Soc. A* **222**, 309-368 (1922).

**Supplementary** Figure S1

**Figure S1. Residuals and the LOWESS curve of the diameter mixed effects models for the estimation dataset.**

**Supplementary** Figure S2

**Figure S2. Residuals and the LOWESS curve of the height mixed effects models for the estimation dataset.**

**Supplementary** MAPLE Code

**Maximum likelihood procedure for fixed effect scenario (bivariate)**

**>restart:**

**with(LinearAlgebra):**

**with(Statistics):**

**with(stats):**

**with(plots):**

**with(Optimization):**

**with(VectorCalculus):**

**with(linalg):**

n := 5220;
M1 := readdata(Estimation);
M2 := convert(M1, matrix);
N := transpose(M2);

Standard errors

Approximated maximum likelihood procedure for mixed effect scenario (bivariate)

restart:

with(LinearAlgebra):

with(Statistics):

with(stats):

with(plots):

with(Optimization):

with(VectorCalculus):

with(linalg):

n := 5220:
M1 := readdata(Estimation):

M2 := convert(M1, matrix):
N := transpose(M2):

**Standard errors**
